# Drug target identification using network analysis: Taking active components in *Sini* decoction as an example

**DOI:** 10.1038/srep24245

**Published:** 2016-04-20

**Authors:** Si Chen, Hailong Jiang, Yan Cao, Yun Wang, Ziheng Hu, Zhenyu Zhu, Yifeng Chai

**Affiliations:** 1 School of Pharmacy, Second Military Medical University, 325 Guohe Road, Shanghai, 200433, China; 2 School of Pharmacy, University of Pittsburgh, 3501 Terrace Street, Pittsburgh, PA, 15261, USA

## Abstract

Identifying the molecular targets for the beneficial effects of active small-molecule compounds simultaneously is an important and currently unmet challenge. In this study, we firstly proposed network analysis by integrating data from network pharmacology and metabolomics to identify targets of active components in *sini* decoction (SND) simultaneously against heart failure. To begin with, 48 potential active components in SND against heart failure were predicted by serum pharmacochemistry, text mining and similarity match. Then, we employed network pharmacology including text mining and molecular docking to identify the potential targets of these components. The key enriched processes, pathways and related diseases of these target proteins were analyzed by STRING database. At last, network analysis was conducted to identify most possible targets of components in SND. Among the 25 targets predicted by network analysis, tumor necrosis factor α (TNF-α) was firstly experimentally validated in molecular and cellular level. Results indicated that hypaconitine, mesaconitine, higenamine and quercetin in SND can directly bind to TNF-α, reduce the TNF-α-mediated cytotoxicity on L929 cells and exert anti-myocardial cell apoptosis effects. We envisage that network analysis will also be useful in target identification of a bioactive compound.

Bioactive compounds exert their biological activities through direct physical binding to one or more cellular proteins^1^. The detection of drug-target interactions is therefore necessary for the characterization of compound mechanism of action^2^. There are two fundamentally different approaches to identify molecular targets of bioactive molecules: direct and indirect^3^. The direct approach utilizes affinity chromatography often with compound-immobilized beads. Many compounds cannot be modified without loss of binding specificity or affinity^4^. Moreover, because of above characteristics, this approach is only suitable to identify targets of one drug once and cannot afford target identification of many compounds simultaneously, such as active components in herbs. With the indirect approach, such as system biology approaches, including proteomics, transcriptomics and metabolomics, are the major tools for target identification and have an unbiased attitude towards all active compounds^5^. A proteomic or transcriptomics approach for identification of binding proteins for a given small molecule or compounds in herbs involves comparison of the protein expression profiles for a given cell or tissue in the presence or absence of the given molecule(s). These two methods have been proved successful in target identification of both many compounds and one drug^6^,^7^,^8^,^9^. Whereas metabolomics has been mainly developed to identify drug(s)-affected pathways^10^,^11^, the “readout”, such as proteins in the pathway, is often far downstream from the drug targets. Therefore using metabolomics for target identification run into the bottleneck.

As bioactive molecules exert their effects through direct physical association with one or more cellular proteins^1^, these target proteins will then act on related proteins, above proteins eventually affect the content of related metabolites. With the advent of the era of big data, now there are large amounts of data about known and predicted protein interactions^12^. Once we use network pharmacology to predict potential targets of active components in Traditional Chinese Medicine (TCM) formula^13^, a component-target protein-related protein-metabolite network can be constructed with the combination of network pharmacology and metabolomics. As a combination of approaches is most likely to bear fruit, the combination of network pharmacology and metabolomics called network analysis could increase the degree of accuracy of target identification of network pharmacology. In addition, metabolomics and network pharmacology employed global profiling methods for the comprehensive analysis of altered metabolites and target proteins, providing insights into the global state of entire organisms, which are well coincident with the integrity and systemic feature of TCM formula. Thus apart from target identification of a bioactive compound, this network analysis method is more beneficial in identifying unknown targets of active compounds in TCM formula simultaneously in an unbiased fashion.

Here, we introduce a new, potentially widely applicable and accurate drug target identification strategy based on network analysis to identify the interactions of active components in TCM formula and target proteins. Our previous studies have confirmed that SND, composed of three medicinal plants: Aconitum carmichaelii, Zingiber officinale and Glycyrrhiza uralensis, can treat heart failure^14^. Metabolomics researches have also been conducted to demonstrate its effectiveness^14^,^15^. Chemome^16^, serum pharmacochemistry^16^ and xenobiotic metabolome^17^ of SND were also characterized. Thus in this study, we took SND as an example to test the potential of network analysis in target identification. Active components in SND against heart failure were identified by serum pharmacochemistry, text mining and similarity match. Their potential targets were then identified by network analysis. At last, the most possible target was validated experimentally to demonstrate the potential of network analysis. Above results will be helpful to investigate the action mechanisms of SND and promote the development of Chinese Drug modernization. More importantly, network analysis will not only conferred a unique advantage to identify targets of active compounds in TCM formula simultaneously, but also provided a new method for the target identification of a bioactive compound. Detailed procedures can be seen in Fig. 1.

## Results

### The rationality of components in SND in absorption and metabolism

Results considering the known metabolism of components in SND have been concluded in Supplementary Table S1. Total flavones (H) and total saponins (Z) were major active components in Glycyrrhiza uralensis. From Table S1, we can conclude that many flavones and saponins in Glycyrrhiza uralensis were known CYP450 inhibitor, while alkaloids in Aconitum carmichaelii are mostly not. Conclusions can be made that Glycyrrhiza uralensis can inhibit the metabolism of alkaloids and improve their bioavailability. Researchers also demonstrated that Glycyrrhiza uralensis can improve bioavailability of diester diterpenoid alkaloids in Aconitum carmichaelli, which coincided with results above. And researchers found that Aconitum carmichaelli can also improve the bioavailability of glycyrrhizic acid which is a major component in Glycyrrhiza uralensis^18^, so we can conclude that the combination of Aconitum carmichaelli and Glycyrrhiza uralensis can enhance efficacy of each medicinal materials. In addition, Zingiber officinale could promote the elimination of diester diterpenoid alkaloids and enhance the absorption of monoester diterpenoid alkaloids^19^. As diester alkaloids are the chief toxic components in Aconitum carmichaelli. The results might be helpful in explaining the mechanism of combination of Aconitum carmichaelli− Zingiber officinale to decrease toxicity and increase efficacy. Researchers also proved that compared with Aconitum Carmichaeli, the bioavailability of three monoester-diterpenoid alkaloids increased in SND^20^. And compared with Aconitum Carmichaeli, the bioavailability of hypaconitine,i.e. diester diterpenoid alkaloids decreased in SND. The SND formula can decrease toxicity and increase efficacy. The information above demonstrated the rationality of components of SND in Absorption and metabolism.

### Compound families and chemical space properties of active components in SND

To give an overview of the compound families in SND, chemical clustering was conducted (Supplementary Fig. S1A). The areas of overlap may show that active components in SND and anti- heart failure drugs are physicochemical property similar, resulting in similar pharmacological actions against heart failure^21^. In addition, the active components are also clustered in three independent areas (region B, C and D), indicating that the active components may be structurally or pharmacologically different in three herbs.

The physicochemical characteristics of a compound are important for its drug likeness. Comparing the physicochemical characteristics of active components in SND with FDA-approved oral drugs will provide insight into the drug likeness of these components. Here, seven physicochemical characteristics of active components in SND were compared with approved orally administered drugs (Supplementary Fig. S1B–H). The overall shapes of the distributions of these characteristics are similar between active components in SND and approved oral drugs, which indicates that many ingredients in herbs have drug potential. The proportion of compounds with more than 10 rotatable bonds in SND is more than in approved drugs (Supplementary Fig. S1G), which means the structures of ingredients in SND are more flexible. There are statistically significant differences between drug and herb of all variables in the aspect of distribution by Kolmogorov-Smirnov test in Supplementary Table S2. As all variables in both drug and herb do not follow normal distributions, we conducted wilcoxon test to evaluate the difference of all variables in drug and herb. The results showed that there are significant differences between drug and herb of all variables in the aspect of median except for Polar Surface Area in Supplementary Table S2. To make a conclusion, physiological characteristics of active components in SND are special while compared with approved oral drugs in the median and distribution.

### Prediction analysis of pharmacological mechanism based on network pharmacology

We constructed the component-target network (Fig. 2A) based on text mining and docking. This network had 109 nodes and 556 edges, in which red circles and hexagons correspond to active components and target proteins, respectively. Many targets in the middle of Fig. 2A are targeted by components in three medicinal herbs, which meant that these targets are main potential targets. According to the data from CHEMBL, BindingDB and PubMed database, 13 out of 61 potential targets were validated to be exact targets of active components in SND (Supplementary Table S3), which proved the reliability of molecular docking and text mining.

The STRING database (version 10.0) (http://string-db.org/) was used to elucidate biological processes, Cellular components, molecular functions and pathways of target proteins. And we only choose meaningful pathways, biological processes, Cellular components and molecular functions with a *p* value < 0.05 as key pathways, processes, components and functions. Functional classification of target proteins is detailed in Fig. 2B. Molecular function of the target proteins is classified to two categories: binding and receptor activity (Fig. 2B). The binding activities that appeared are mainly associated with receptor binding and enzyme binding. And the receptor activity is adrenergic receptor activity. According to the classification of cellular component, the proteins are located in cytoplasmic part, extracellular region and membrane region. The biological processes that the target proteins are involved can be summarized in Fig. 2B. The results firstly demonstrated that SND exerted its protective effects by regulation of blood circulation^22^, response to oxidative stress^23^,^24^, regulation of apoptotic process^25^ and inflammatory response^26^, which coincided with previous researches. In addition, the results also indicated that active components in SND could also exert anti- heart failure effect through regulation of blood pressure, regulation of vasodilation, regulation of muscle contraction, regulation of heart contraction, blood coagulation and regulation of angiogenesis. Although large amounts of references showed that the above six processes were closely related to heart failure^27^,^28^,^29^,^30^,^31^,^32^, further experiments are needed to identify the relationship between SND and these six biological processes.

To find the relations between target proteins and the important pathway further, we constructed the target-pathway network (Fig. 3A) based on the data extracted from STRING. There were several target proteins in one pathway and one target protein always existed in many pathways. Logically, the role of one pathway which contain many target proteins that interacted with drug molecules is more vital than the role of one target protein that interacted with drug molecules in many pathways, which is because that the impact of one target protein on the whole pathway maybe little, and the impact of a pathway which contained many target proteins interacted with the drugs on the body could be huge. Therefore, we tried to find the most important pathways through analyzing the target –pathway network. The pathways related with target proteins can be summarized in Fig. 3A. These results firstly demonstrated that SND exerted its protective effects against heart failure primarily by regulating above 15 pathways. Previous studies have demonstrated that SND exerted its cardiotonic effect by regulation of TNF signaling pathway^33^, Hypertrophic cardiomyopathy (HCM)^22^, PI3K-Akt signaling pathway^34^ and Dilated cardiomyopathy^23^,^35^. Although large amounts of references showed that heart failure was closely related to HIF-1 signaling pathway^36^, Calcium signaling pathway^37^, cGMP-PKG signaling pathway^38^, mTOR signaling pathway^39^, Renin-angiotensin system^40^, ErbB signaling pathway^41^, AMPK signaling pathway^42^, VEGF signaling pathway^43^, Vascular smooth muscle contraction^44^, Adrenergic signaling in cardiomyocytes^45^ and Estrogen signaling pathway^46^, further experiments are still needed to identify the relationship between SND and these 11 pathways. In order to further explore the possible mechanism of active components in SND, we classified the targets protein into three parts according their degree in drug-target network: high degree (20–41), middle degree (10–19) and low degree (1–9). Then the *p* value of every relevant pathway was calculated in the three parts (Supplementary Table S4). We only considered pathways with a *p* value < 0.05. Lower *p* values represent that pathways have higher amounts of proteins involved in, and were meaningful in the global pathways. Comparing the results of three parts we found that high degree and low degree targets are mainly related with HIF-1 signaling pathway and Calcium signaling pathway, whereas middle degree targets are primarily bound up with Dilated cardiomyopathy and TNF signaling pathway.

In summary, SND exerted its protective effects mainly by regulating 10 biological processes and 15 pathways. In addition, references have demonstrated that SND can cure heart failure by regulation of blood circulation, response to oxidative stress, apoptotic process, inflammatory response, TNF signaling pathway, Hypertrophic cardiomyopathy, PI3K-Akt signaling pathway and Dilated cardiomyopathy. Another six biological processes and 11 pathways which heart failure involves in, such as regulation of vasodilation, regulation of muscle contraction, HIF-1 signaling pathway, Calcium signaling pathway and so on, are also identified to be regulated by SND to exert its anti- heart failure effects and needed to be verified by experiments. The multiple active components in SND can target the multiple proteins in the biological network to regulate and restore the network equilibrium, thereby controlling the occurrence and development of heart failure.

### Construction and analysis of target protein-disease networks

Then we analyzed the category of the targets related diseases, the results were listed in Fig. 3B. We found that cancer, immune diseases, cardiovascular diseases, endocrine and metabolic diseases and infectious diseases were the main disease category. Though the proportion of cardiovascular diseases is not high, there were large amounts of experimental results reported that the SND can cure some cardiovascular diseases, such as heart failure^14^, myocardial infarction^47^, etc. Fig. 3B also indicated that SND can cure some liver diseases, such as non-alcoholic fatty liver disease and hepatitis B, which is in accordance with previous researches^48^. In addition, researches demonstrated that herbs in SND can cure type 2 diabetes mellitus^49^ and rheumatoid arthritis^50^. But the experimental reports of cancer and parasitic infectious diseases were seldom, it may be due to the complex mechanism of those diseases and the different ratio of the ingredients in SND.

### Network analysis for identification of targets of active components in SND

To reduce the number of candidate targets and identify more potential targets based on targets identified from network pharmacology, a component –protein – metabolite interaction network (CPMI) was established through the integration of NATPI and MPPI, as shown in Fig. 4. This network consists of chemical components, target proteins, pathway proteins, and biomarkers, including 130 nodes and 375 edges. The components acting on target proteins would cause the up- or down- regulation of related metabolic enzymes in pathway, thereby resulting in the changes in concentration of biomarkers. The interactions between target proteins and pathway proteins were obtained from STRING with a high confidence (>0.8). We consider the active components as the initial node and the metabolites as the terminal node. As shown in Fig. 4, 48 components interacted with 25 target proteins. These proteins can be related to 13 biomarkers through 44 pathway proteins. In this network, 25 target proteins could be more likely to be the true targets of active components in SND compared to 61 target proteins identified from network pharmacology. This deduction has been proved by references. The detailed evidence is that a total of 61 putative target proteins were identified by network pharmacology, only 13 target proteins were validated by reference. While among 26 target proteins identified by network analysis, nine target proteins are in agreement with existing research results. Target proteins of active components validated by references were summarized in supplementary data (Supplementary Table S3).

Then we took Angiotensin II Receptor (Type 1) (AGTR1), TNF-α and Heme oxygenase 1 (HMOX1) as an example to explain the potential synergistic mechanism of active components in SND for curing heart failure (Fig. 4). Researches showed that in the heart, apoptosis and skeletal muscle atrophy can be triggered by angiotensin II^51^. And many authors have suggested that AGTR1 stimulation brought about apoptosis and skeletal muscle atrophy^51^. In contrast, an AGTR1 blocker can block apoptosis and skeletal muscle atrophy to cure heart failure^52^. As AGTR1 has been identified and validated to be a target of active components in SND (Fig. 4), we can deduce that these components prevent apoptosis and skeletal muscle atrophy to cure heart failure through blocking AGTR1.

Evidence shows that TNF-α is capable of modulating cardiovascular functions through a variety of mechanisms such as inducing left ventricular dysfunction, left ventricular remodeling, abnormalities in myocardial metabolism, cachexia, uncoupling of β-receptor from adenylate cyclase and triggering platelet activation^53^. Further, TNF-α is involved in the production of other inflammatory cytokines like IL-6 and IL-1 which enhance TNF-α-induced myocardial depression and cytotoxicity^53^. Moreover, TNF-α triggers apoptosis in cardiomyocytes through activation of neutral sphingomyelinase pathway and apoptosis may be a major reason for progressive cardiac dysfunction in human heart failure^53^. If TNF-α is a detrimental factor or mediator for myocardial failure, inhibition of TNF-α either in blood or in TNF-α receptors may be an effective treatment for heart failure. However, clinical trials of TNF-α antagonists for curing heart failure failed. Reasons can be concluded as the following: Firstly, protective effect of NF-κB had been reported in reovirus-infected myocarditis, complete inhibition of TNF-α may lose the benefit of NF-κB activation^54^; Secondly, tumor necrosis factor-alpha confers cardioprotection, complete inhibition of TNF- α may be a detrimental factor for failing hearts^55^. A conclusion can be made that TNF- α antagonists can be an effective treatment for heart failure, whereas complete inhibition of TNF-α may be a detrimental factor for failing hearts^56^. Because of the moderate activity of small molecules, we can deduce that these molecules may prevent apoptosis, left ventricular dysfunction, left ventricular remodeling and so on to cure heart failure through targeting TNF-α. As TNF-α has been identified to be a target of active components in SND, these components may confer cardioprotection by targeting TNF-α.

HMOX1 is an inducible stress-response protein that imparts antioxidant and anti-apoptotic effects^57^. HMOX1 induction in the failing heart is an important cardioprotective adaptation that opposes pathological left ventricular remodeling, and this effect is mediated, at least in part, by carbon monoxide (CO)-dependent inhibition of mitochondrial permeability transition and apoptosis^58^. Augmentation of HMOX1 or its product, CO, may represent a novel therapeutic strategy for ameliorating heart failure^58^. As HMOX1 was identified to be a target of active components of SND, these components may treat heart failure through opposing pathological left ventricular remodeling caused by HMOX1 induction.

To make a conclusion, active components in SND exert their effects against heart failure by AGTR1, TNF-α and HMOX1 simultaneously. The detailed mechanism is that these components can prevent apoptosis, skeletal muscle atrophy, left ventricular dysfunction, pulmonary oedema, left ventricular remodeling and so on to cure heart failure through targeting above three targets.

### Experimental target validation

TNF-α was firstly validated experimentally for three reasons. First, TNF-α was found both by dock and text mining in the middle of Fig. 2A. Second, TNF-α was in the center of target protein–target protein interaction networks (supplementary Fig. S2). And there is ample literature support for the important role of TNF-α in heart failure^59^,^60^. Last but not least, our above pathway enrichment analysis of target proteins and previous researches both demonstrated that SND exerted its cardiotonic effect by regulation of TNF signaling pathway^33^, Apoptosis^35^, Hypertrophic cardiomyopathy (HCM)^22^ and Dilated cardiomyopathy^23^,^35^, and TNF existed in above four pathways simultaneously. Thus we inferred that TNF could be the main targets of active components in SND.

Total alkaloids, total gingerols, total flavones and total saponins are the main active components of each single-herb of SND responsible for curing cardiovascular diseases respectively^15^. Total alkaloids can traditionally be divided into three major types: diester diterpenoid alkaloids, monoester diterpenoid alkaloids and alkylolamine diterpenoid alkaloids^61^. Aconitine, hypaconitine and mesaconitine are representative components of diester diterpenoid alkaloids from Chinese pharmacopoeia. Higenamine, hypaconine, mesaconine and talatisamine are major reported active components of alkylolamine diterpenoid alkaloids. Talatisamine is found and validated to be an anti-heart failure compound in our previous study^62^. Glycyrrhizic acid and quercetin is major components of total saponins and total flavones respectively. 6-Gingerol is a representative component of total gingerols. Because of the representation of above ten compounds, we choose them to test the interaction between active components in SND and TNF-α.

### Active components in SND inhibits TNF-α–mediated cytotoxicity on L929 cells

We began our validation of interaction between active components in SND and TNF-α using TNF-α-mediated L929 cytotoxicity assay. Results showed that SND, hypaconitine, mesaconitine, higenamine and quercetin are effective small molecule inhibitors of TNF-α. As shown in Fig. 5, SND inhibits TNF-α-mediated cytotoxicity on L929 cells dose-dependently within the range of 0.39–12.5 mg/ml. In addition, hypaconitine, mesaconitine, higenamine and quercetin inhibit TNF-α-mediated cytotoxicity on L929 cells dose-dependently within the range of 6.75–200 μM. Whereas, aconitine, 6-gingerol and glycyrrhizic acid are not effective small molecule inhibitors of TNF-α.

### Active components in SND directly binds to TNF-α

We next investigated whether active components in SND targeted TNF-α with SPR analysis. As shown in Fig. 6F, SND (8.75–140 mg/ml) could bring about a concentration-dependent resonance change when flowing through the sensor chip coated with TNF-α, indicating the direct binding of active components in SND to TNF-α. In addition, hypaconitine, mesaconitine, higenamine and quercetin (6.25–400 μM) also brought about a concentration-dependent resonance change when flowing through the sensor chip coated with TNF-α, indicating the direct binding of hypaconitine, mesaconitine, higenamine and quercetin to TNF-α (Fig. 6). The equilibrium dissociation constant (K_D_) was calculated to be 53 μM, 57.5 μM, 67 μM and 35 μM. These results are consistent with results of TNF-α–mediated cytotoxicity on L929 cells, which demonstrated that SND, hypaconitine, mesaconitine,higenamine and quercetin are effective small molecule inhibitors of TNF-α. This provided us with a set of leads against TNF-α belonged to diterpenoid alkaloids.

### The protective effect of active components in SND on DOX-induced injury of cardiomyocytes

We then investigated whether these small molecule inhibitors of TNF-α have cardiotonic effect. As shown in Fig. 7, SND, aconitine, hypaconitine, mesaconitine and higenamine (6.25–200 μM) alone had a slight promoting proliferation but there were no significant differences from the control group (*p* > 0.05). Whereas quercetin alone had a promoting proliferation and there are significant differences from the control group (*p* < 0.05). To analyze the effects of SND, aconitine, hypaconitine, mesaconitine, higenamine and quercetin (6.25–200 μM) on DOX-induced cytotoxicity in H9C2 cells, cell viability was examined after incubation with these compounds in the presence of DOX (2 μM). Above compounds pretreatments all provided good protective effects on DOX-mediated cell death in a dose-dependent manner (*p* < 0.05, compared to DOX group) (Fig. 7). These results confirmed the cardioprotective effect and noncytotoxicity of these six compounds *in vitro*.

In conclusion, we verified the direct inhibition of SND, hypaconitine, mesaconitine, higenamine and quercetin on TNF-α in molecular and cellular level, and examined the protective effect of SND, hypaconitine, mesaconitine, higenamine and quercetin on DOX-induced injury in cardiomyocytes. In addition, although aconitine, 6-gingrol and glycyrrhizic acid are not effective small molecule inhibitors of TNF-α, they have been demonstrated to have anti-heart-failure activity^63^,^64^. We can draw the conclusion that there existed other target proteins of active components in SND, which coincided with the synergistic effect theory of multi-components and multi-targets of traditional Chinese medicine formula.

## Discussion

In principle, Network analysis has the potential to allow the target identification of both bioactive compounds in medicinal herbs simultaneously and an active compound resulting from phenotypic screens. Although network pharmacology and metabolomics have been applied to target identification before^65^,^66^, the combination of them for target identification is novel. Furthermore, because the global profiling characteristics of metabolomics and network pharmacology are well coincident with the integrity and systemic feature of TCM formula, the main advantage of our network analysis method is identifying unknown targets of active compounds in TCM formula simultaneously in an unbiased fashion, which will promote the clarification of mechanism of TCM formula. We have established here that network analysis is a potentially applicable and accurate drug target identification strategy.

A key requirement for the success of network analysis is that active compounds must have clear efficacy in the therapy of detailed diseases. In addition, there must exist many known targets of the disease. Network analysis is complementary to existing chemical genetic assays for drug target identification. Although in some cases chemical genetic assays can identify a list of genes as targets, which could be a mixture of true direct targets and proteins involved in the same or parallel biological pathway(s), the network analysis discussed here might be able to identify the targets interacting directly with treated diseases. The combined methods together can provide a common ground for target elucidation, validation, and characterization, and contribute to our understanding of biological pathways and networks affected by bioactive compounds.

Ultimately, the identification of low abundance, weakly bound targets or membrane proteins is a challenge in target identification^1^. Examples we show in this study demonstrate that our network analysis can address these factors and can identify targets in an unbiased way. Network analysis is applicable to characterization of chemical probes resulting from phenotypic screens and can potentially provide useful information regarding drug mechanism of action in a systematic, hypothesis generating way. In principle, its applicability extends beyond small compounds, to include any monitor the interaction of other ligands, such as peptides or even antibodies, with proteins.

Network pharmacology-based approach is to use computational methods to find putative binding proteins for a given compound. Reverse docking is one main computational approach. This approach is to dock a compound with a known biological activity into the binding sites of three-dimensional (3D) structures of a given protein. However, reverse docking still has certain limitations. The major one is that the protein entries in the protein structure databases, like the PDB, are not enough for covering all the protein information of disease-related genomes. The second one is that this approach has not considered the flexibility of proteins during docking simulation, and this aspect will produce negative false. Another limitation is that the scoring function for reverse docking is not accurate enough, which will produce positive false^67^. Our tendency to overcome these shortages in this study is to integrate this method with text mining and metabolomics. The integration of these methods is called network analysis. In summary, the applicability of network analysis is currently limited to active compounds with known diseases, and to complex diseases with multiple targets. In addition, because of the limitation of detection in analytical tools such as LC/MS, GC/MS and so on, not all metabolite biomarkers with changed content can be detected, which could be the reason why only nine out of 13 target proteins exist in network analysis.

In this study, serum pharmacochemistry, text mining and similarity match were firstly used to identify 48 potential anti- heart failure components in SND. And 61 potential targets of these components were identified by network pharmacology. Biological process and pathway enrichment analysis of these targets demonstrated that SND could exert cardiotonic effect by regulating 10 biological processes and 15 pathways. Among them, four biological processes and four pathways had been validated to be regulated by SND to exert its effect in previous researches. Based on results of network pharmacology, network analysis was further conducted to identify more potential targets of active components in SND, which leaded to a decrease in the number of targets from 61 to 26. Previous researches demonstrated that nine out of 26 targets had been verified by references, while only 13 out of 61 targets were verified by references. And among the targets predicted by network analysis, TNF-α was firstly experimentally validated to be the targets of active components in SND in molecular and cellular level. Above results indicated that network analysis is precise in target identification. This method will be helpful to investigate the mechanisms of TCM formula and promote the development of Chinese Drug modernization.

## Materials and Methods

### Analyzing the absorption and metabolism of components in SND

SND is composed of three medicinal plants: Aconitum carmichaelii, Zingiber officinale and Glycyrrhiza uralensis. Extensive studies have shown that total alkaloid (S), total gingerols (J), total flavones (H) and total saponins (Z) are the main active components of each single-herb of SND responsible for curing cardiovascular diseases respectively^15^,^16^. Thus 43 alkaloids (S), 64 gingerols (J), 60 flavones (H) and 29 saponins (Z) of SND were collected as the potential main active components in our previous study^15^. And the chemical information of these components (structure, canonical name, and CID number) employed for computational analysis have also been collected. In order to evaluate the rationality of SND in absorption and metabolism, we firstly searched the public available database admetSAR (http://lmmd.ecust.edu.cn:8000/) using similarity search. Results considering the known absorption and metabolism of 196 components were found.

### Construction of a chemical database of anti-heart failure components in SND

We identified anti-heart failure components database of SND following the three steps. Firstly, our previous studies have analyzed the components and metabolites of SND in plasma and urine using UPLC/Q-TOF-MS^16^,^17^. It’s generally accepted that compounds from herbal medicines that appear *in vivo* have a chance to exert their effects^68^, thus we added the prototype compounds and their metabolite *in vivo* into the previous constructed chemical database of SND^15^. To make a conclusion, we have collected 196 *in vitro* components and 112 *in vivo* components in SND. The detailed information on these *in vivo* components is described in the supplementary data (Table S5). Secondly, as the main components of three herbs in SND have been isolated and tested for different kinds of activity during the past years^63^,^69^, text mining can dig out most of the primary active components. We filtered active components from the article databases (Chinese Pharmacopoeia 2010 edition, web of science, http://www.wanfangdata.com.cn/, http://www.cnki.net/, and www.ncbi.nlm.nih.gov/pubmed/) based on above *in vivo* and *in vitro* components; Due to the limitation of UPLC/Q-TOF-MS method in detecting trace substances in biological sample, some *in vivo* components in SND cannot be identified in our previous papers. Thus, both the *in vivo* and *in vitro* components were taken into consideration in the process of text mining. Thirdly, for unreported components in SND with potential anti-heart failure activity, drug similarity search tool in Therapeutic Targets Database (TTD, http://xin.cz3.nus.edu.sg/group/cjttd/ttd.asp) was used to screen the similar drugs of these components through the structural similarity comparison. TTD provides comprehensive information about efficient targets and the corresponding approved, clinical trial, and investigative drugs. All information provided in TTD is fully referenced. We only selected the drugs with high similarity score (≥0.85) in comparison with the structures of components in SND in order to get a more accurate results. The therapeutic targets of these similar drugs were also collected as predicted effector molecules of SND. If the targets are associated with heart failure, the related components will be considered as potential active ingredients. In total, we obtained 48 active components of SND. The detailed information on these active components is described in Table 1.

### Chemical space mapping and analysis

In order to investigate whether active anti-heart failure components in SND and anti-heart failure drugs had similar physicochemical properties, the physicochemical properties of 48 active components and 71 drugs collected in drugbank using the keywords “heart failure” were calculated using commercial software Discovery Studio 2.5 (http://www.accelrys.com). The properties included molecular weight, the number of aromatic rings, the number of hydrogen bond donors, the molecular polar surface area, the number of rotatable bonds, ALogP, the number of hydrogen bond acceptors. Distribution of these compounds in the chemical space was visualized via principal component analysis using SIMCA-P V 13.0 (demo, Umetrics, Sweden). In addition, as physicochemical characteristics of a compound are also important for its drug likeness. Comparing the physicochemical characteristics of active components in SND with FDA-approved oral drugs will provide insight into the drug likeness of these ingredients. We collected 105 approved oral drugs from drugbank, and the same seven properties were calculated in the same way as above descriptions.

### Target identification

There are two methods for identification of potential targets of active components in SND as follows. First, true targets of 71 FDA-approved anti-heart failure drugs were retrieved from DrugBank database in 2015. And proteins associated with heart failure were also considered as potential targets of heart failure by searching OMIM ((http://www.ncbi.nlm.nih.gov/omim/), UniProtKB (http://www.uniprot.org/), TTD and GeneCards (http://www.genecards.org/) databases in 2015 using the following search terms: heart failure, cardiac failure, cardiac dysfunction, cardiac insufficiency, cardiomyopathy, ventricular dysfunction, chronic heart failure, congestive heart failure, heart insufficiency and heart decompensation. And we have deleted the false positives that are not related with heart failure during the process of text mining. The quality control statistics on the performance of text mining were shown in Supplementary Table S6. Molecular docking between active components and heart failure related target proteins was conducted using libdock in Discovery Studio 2.5 or Autodock vina^70^. All the protein structures were directly downloaded from the RCSB protein data bank (www.pdb.org) with their resolutions being carefully checked. The co-crystallized molecules binding with target proteins were regarded as positive drug. The dock scores of positive drugs with corresponding proteins were defined as cutoff value. If the dock score of a component with relavant protein is higher than the cutoff value, this protein was then considered as a potential target protein of this component. Second, the targets of components were searched in Herbal Ingredients’ Target (http://lifecenter.sgst.cn/hit/), TargetHunter Database (http://​www.​cbligand.​org/​TargetHunter), TCMID (http://www.megabionet.org/tcmid/) and CHEMBL (https://www.ebi.ac.uk/chembl/) by the structure of components. The potential targets of components gained by above method were further searched in TTD, PharmGkb (www.pharmgkb.org) and DrugBank (http://www.drugbank.ca/). We only retain targets which have relationships with heart failure. As a result, an active component–target protein interaction network (CTPI) can be constructed with the results from above two methods and displayed by Cytoscape 3.0 71. A component and a related potential target protein can be linked with an edge.

### Pathway enrichment analysis of metabolite biomarkers and network construction

A comprehensive tool suite for metabolomics data analysis MetaboAnalyst 3.0 (http://www.metaboanalyst.ca/) was used to enrich the pathway of metabolite biomarkers from previous study. The proteins in the enrichment pathways (*p* < 0.05) were extended to their nearest neighbors, and subsequently, a metabolite–pathway protein interaction network (MPPI) was constructed by Cytoscape 3.0.

### Network Analysis

**Figure 1:**
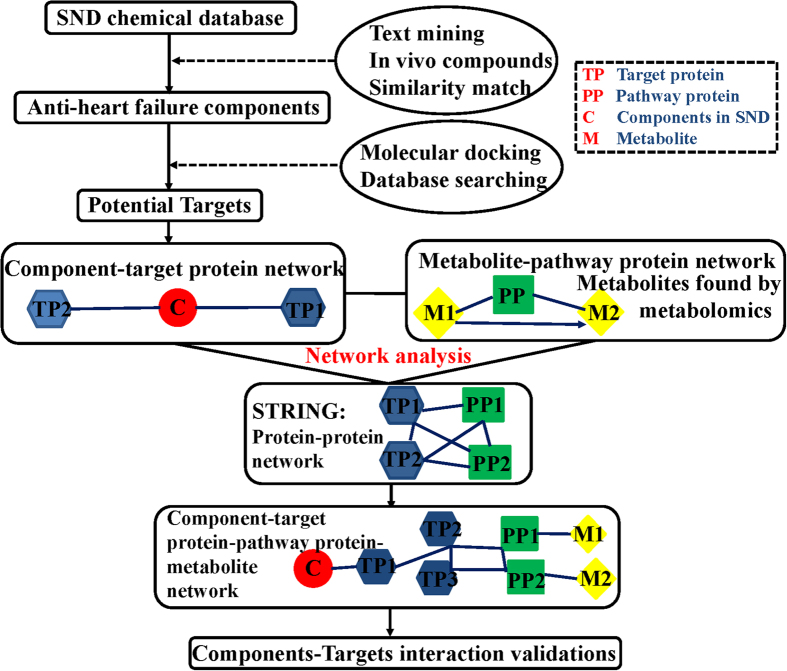
The flowchart of network analysis approach.

**Figure 2:**
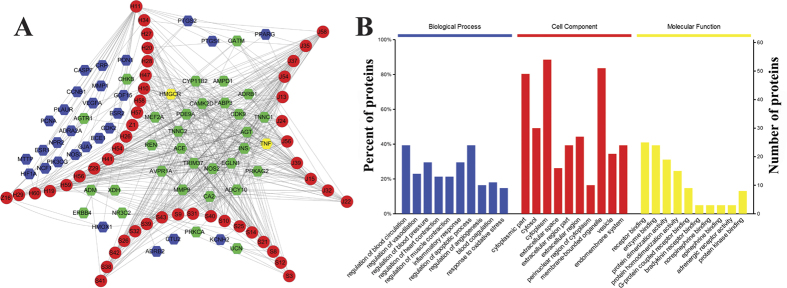
(**A**) The Component-Target network. The red circles represent the 48 active components in SND, S, J, H and Z refers to alkaloids, gingerols, flavones and saponins in Aconitum carmichaelii, Zingiber officinale and Glycyrrhiza uralensis. The blue hexagons represent the gene names of targets of the three herbs found by text mining, while the green hexagons are the targets found by dock. The yellow hexagons represent the gene names of targets found both by text mining and dock. Targets in the center of network represent the common targets of three herbs, and targets in the curve of S, J or H and Z represent the targets of each kind of active components respectively. (**B**) The enrichment analysis in biological processes, cellular components and molecular functions of 61 identified target proteins by STRING database.

**Figure 3:**
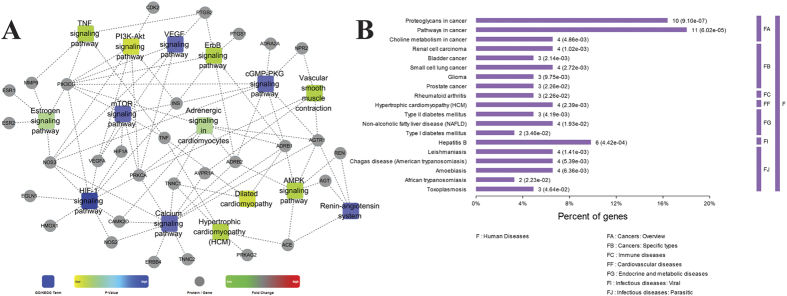
(**A**) The Target-Pathway network by STRING database. (**B**) The enrichment analysis of diseases of these target proteins by STRING database.

**Figure 4:**
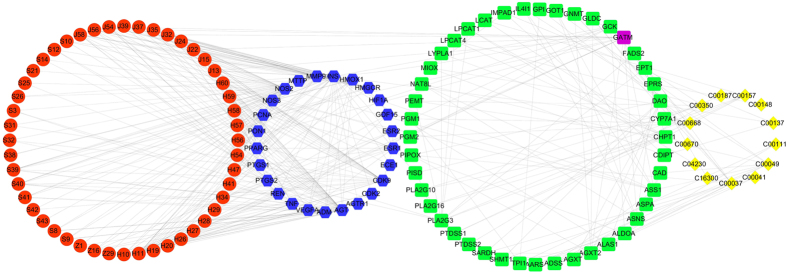
Component – protein–metabolite network. The active components, target proteins, pathway proteins and metabolites are represented by the red circles, blue hexagons, green round rectangles, and yellow diamonds, respectively. GATM represented by a purple round rectangle is both target protein and pathway protein. The interactions between active components and proteins, between proteins and proteins and between proteins and metabolites are linked by edges, respectively.

**Figure 5:**
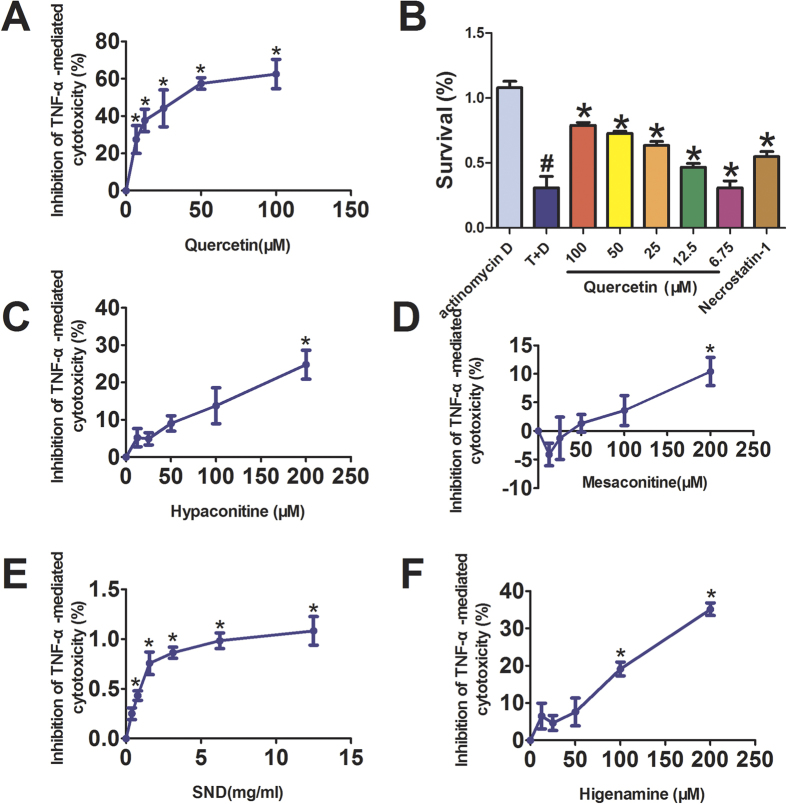
Active components in SND inhibited TNF-α-mediated cytotoxicity on L929 cells. L929 cells were treated for 18 h with 10 ng/ml of TNF-α and 1 mg/ml of Actinomycin D (**D**) in the presence of indicated concentrations of active components. TNF-α-mediated cytotoxicity on L929 cells were measured with CCK8 assay. Data were obtained from three independent experiments performed in triplicate and presented as means (±SD). *p < 0.05 vs. TNF-α (T) only, ^#^*p* < 0.05 vs. Actinomycin D (**D**) only. Necrostatin-1 (20 μM) is used as a positive drug.

**Figure 6:**
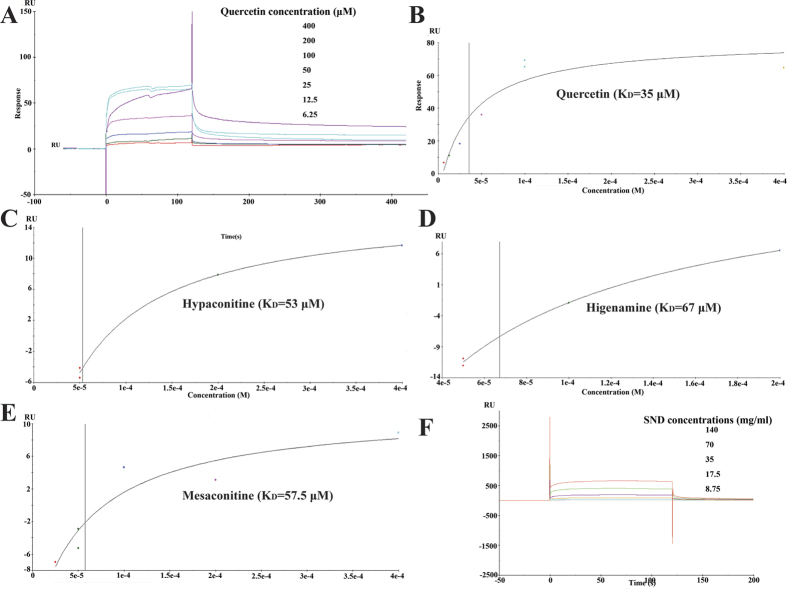
SPR analysis showed that quercetin, hypaconitine, mesaconitine, higenamine and SND directly bound to TNF-α. (**A**) The sensorgrams indicate the direct binding of quercetin to TNF-α immobilized on a CM5 sensor chip. The kinetic measurements were performed in triplicate using a set of serial dilutions as shown. (**B**–**E**) Standard curves and K_D_ of quercetin, hypaconitine, mesaconitine and higenamine. (**F**) The sensorgrams indicate the direct binding of SND to TNF-α immobilized on a CM5 sensor chip. The kinetic measurements were performed in triplicate using a set of serial dilutions as shown. Data in (**A**–**F**) were representatives of three independent experiments.

**Figure 7:**
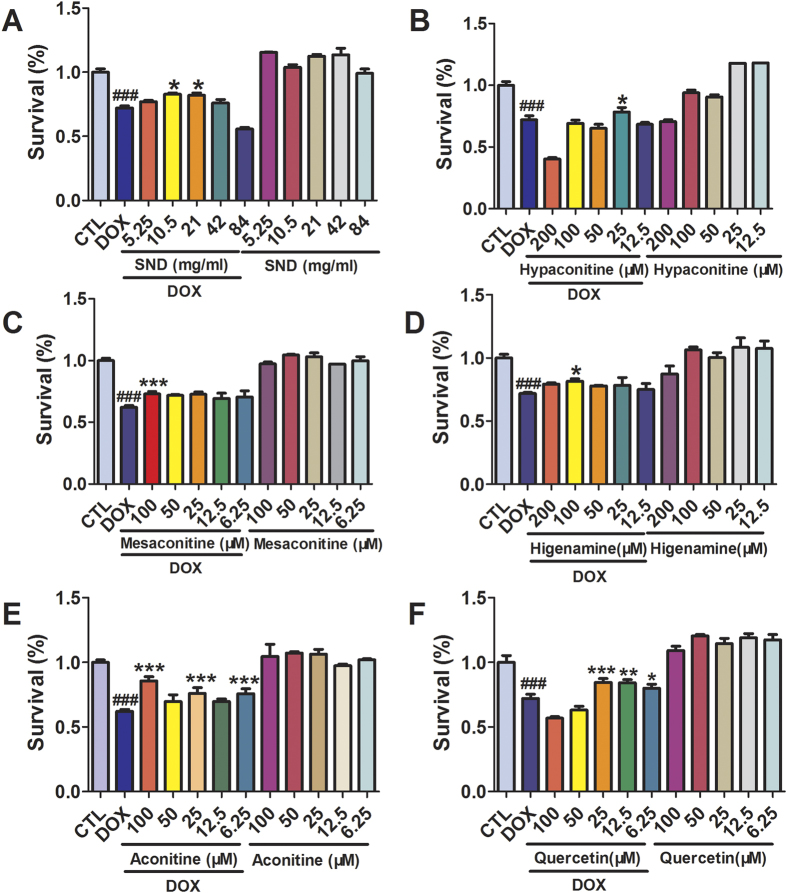
The active components in SND protected Rat cardiac H9C2 cell line from DOX-induced cell death. Cells were incubated with active components in SND (6.25–100 μM) in DMEM supplemented with 0.5% fetal bovine serum at 37 °C for 2 h followed by incubation with or without DOX (2 μM) for another 24 h. Cell viability was determined by CCK8 assay. Results were expressed as percentages of control group. Data are shown as mean ± SD from three independent experiments. ^###^*p* < 0.001 DOX group versus control group, *p < 0.05, **p < 0.01, ***p < 0.001 treatment group versus DOX group.

According to the collected candidate genes and proteins related to heart failure, a protein–protein interaction network (PPI) was built by Cytoscape 3.0. Subsequently, PPI combined with CTPI was used to establish a new active component – target protein interaction network (NATPI). NATPI combined with MPPI was then used to establish a component – protein–metabolite interaction network (CPMI). Above process called network analysis can identify targets which can connect components with different metabolites regulated by these components, detailed procedures can be seen in Fig. 1. With the combination of metabolomics and network pharmacology, obviously target proteins identified by network analysis will be more precise than network pharmacology alone. To facilitate scientific interpretation of identified potential targets, several analyses were performed. First, based on the potential targets, gene ontology (GO) enrichment analysis and pathway enrichment analysis were utilized to identify related molecular functions, involved biological processes, cellular components and pathways by STRING. Second, enrichment of target related diseases was also conducted by STRING.

### Experiments for verification

Aconitine, Hypaconitine, Mesaconitine, Higenamine, Hypaconine, Mesaconine, Talatisamine, Glycyrrhizic acid, Quercetin and 6-Gingerol (purity 99%) were purchased from Shanghai standard Corporation (http://naturestandard.cn.alibaba.com). The structures of above chemicals were unambiguously identified by ^1^H NMR and MS spectra, and their purity were over 99% determined by HPLC-UV. Recombinant mouse TNF-α were purchased from Sigma (St.Louis, MO, USA). Actinomycin D was purchased from Sigma (St.Louis, MO, USA). Necrostatin-1 was purchased from Selleck Chemicals (Houston, USA). L929 mouse fibroblast cell line and Rat cardiac H9C2 cell line were obtained from Cell Bank of the Chinese Academy of Sciences (Shanghai, China). Dulbecco minimal essential medium (DMEM) was purchased from Invitrogen Corporation (Grand Island, NE, USA) and supplemented with 10% fetal calf serum (FBS) obtained from Gibco Co. (Rockville, MD, USA). Dimethyl sulfoxide (DMSO), penicillin streptomycin, and trypsin were purchased from Gibco Co. All experiments were repeated three times.

### Assay for TNF-α mediated L929 cytotoxicity

TNF-α mediated L929 cytotoxicity was copied as described previously^72^. Different amounts of aconitine, hypaconitine, mesaconitine, higenamine, hypaconine, mesaconine, talatisamine, glycyrrhizic acid, quercetin and 6-gingerol (12.5–200 μM) were mixed with 10 ng/ml TNF-α and 1 μg/ml Actinomycin D applied to the cells. Necrostatin-1 (20 μM) was used as a positive drug. After 18 h incubation, cell viability was assessed by microscope examination and CCK8 assay. The optical density (OD) was measured at 450 nm in a microplate reader (Synergy^TM^ 4, BioTek, USA). The percentage inhibition of cytotoxicity was calculated using the following formula: (OD_actinomycinD+TNF-a+components_ − OD_actinomycinD+TNF-α_)/(OD_actinomycinD_ − OD_actinomycinD+TNF-α_) × 100.

### Surface plasmon resonance (SPR) analysis

SPR measurements were performed on a BIAcore T200 instrument (GE Healthcare, Little Chalfont, Buckinghamshire, United Kingdom) at 25 °C using PBS with 5% DMSO as running buffer with a constant flow rate of 30 ml/min. 100 mg/ml TNF-α in 10 mM sodium acetate buffer (pH 5.0) was covalently immobilized onto the CM5 sensor chip (GE Healthcare) using standard primary amine coupling procedure. Gradient concentrations of components (6.25–400 μM) dissolved in the running buffer were injected into the channel for 60 s, followed by disassociation for 120 s. The data were analyzed with the BIAevaluation 3.0 software (BIAcore) using a 1:1 binding model.

### Assay for Doxorubicin (DOX) mediated H9C2 cytotoxicity

DOX mediated H9C2 cytotoxicity was also copied as described previously^73^. Cells were incubated with aconitine, hypaconitine, mesaconitine, higenamine, hypaconine, mesaconine, glycyrrhizic acid, quercetin and 6-gingerol (6.25–100 μM) in DMEM supplemented with 0.5% fetal bovine serum at 37 °C for 2 h followed by incubation with or without DOX (2 μM) for another 24 h. Cell viability then was tested by CCK-8 (Beyotime Biotechnology, Jiangsu, China).

### Data analysis

All quantitative values are given as means (±SD). Statistical analysis was performed using one-way analysis of variance (ANOVA) test, followed by Dunnett’s multiple comparison post hoc test. *p* < 0.05 was considered to be statistically significant.

## Additional Information

**How to cite this article**: Chen, S. *et al.* Drug target identification using network analysis: Taking active components in *Sini* decoction as an example. *Sci. Rep.* **6**, 24245; doi: 10.1038/srep24245 (2016).

## Supplementary Material

Supplementary Information

## Figures and Tables

**Table 1:** Potential active components in SND.

No	Name	Formula	Serial number
**1**	14-Acetyltalatizamine^a^	C26H41NO6	S14
**2**	6-Gingerol^a^	C17H26O4	J54
**3**	Daidzein^a^	C15H10O4	H60
**4**	Formononetin^a^	C16H12O4	H34
**5**	Fuziline^a^	C24H39NO7	S9
**6**	Gancaonin L^a^	C20H18O6	H41
**7**	Glycycoumarin^a^	C21H20O6	H54
**8**	Glycyrrhizin^a^	C42H62O16	Z16
**9**	Hetisine^a^	C20H27NO3	S42
**10**	Hypaconine^a^	C24H39NO8	S8
**11**	Hypaconitine^a^	C33H45NO10	S26
**12**	Isoliquiritigenin^a^	C15H12O4	H29
**13**	Licochalcone D^a^	C21H22O5	H58
**14**	Licoisoflavone^a^	C20H18O6	H59
**15**	Liquiritigenin^a^	C15H12O4	H20
**16**	Mesaconine^a^	C24H39NO9	S3
**17**	Neoline^a^	C24H39NO6	S10
**18**	Talatisamine^a^	C24H39NO5	S12
**19**	Glycyrrhetic acid^a^	C30H46O4	Z29
**20**	Aconitine^a^	C34H47NO11	S25
**21**	Mesaconitine^a^	C33H45NO11	S21
**22**	Davidigenin^b^	C15H14O4	H56
**23**	4-Shogaol^c^	C15H20O3	J32
**24**	8-Gingerol^c^	C19H30O4	J56
**25**	8-Paradol^c^	C19H30O3	J24
**26**	8-Shogaol^c^	C19H28O3	J37
**27**	10-Gingerol^c^	C21H34O4	J58
**28**	10-Shogaol^c^	C21H32O3	J39
**29**	1-Dehydro-10-gingerdione^c^	C21H30O4	J15
**30**	1-Dehydro-6-gingerdione^c^	C18H26O3	J13
**31**	6-Paradol^c^	C17H26O3	J22
**32**	6-Shogaol^c^	C17H24O3	J35
**33**	Beiwutinine^c^	C23H37NO10	S43
**34**	Coryneine^c^	C11H18NO2	S32
**35**	Echinatin^c^	C16H14O4	H28
**36**	Fuzinoside^c^	C15H28O13	S40
**37**	Glabridin^c^	C20H20O4	H47
**38**	Glycyrrhizic acid^c^	C42H62O16	Z1
**39**	Higenamine^c^	C16H17NO3	S39
**40**	Licochalcone A^c^	C21H22O4	H26
**41**	Licochalcone B^c^	C16H14O5	H27
**42**	Licochalcone C^c^	C21H22O4	H57
**43**	Neolinine^c^	C23H37NO6	S41
**44**	Pinocembrin^c^	C15H12O4	H19
**45**	Quercetin^c^	C15H10O7	H11
**46**	Rutin^c^	C27H30O16	H10
**47**	Salsolinol^c^	C10H13NO2	S31
**48**	Songoramine^c^	C22H29NO3	S38

^a^*In vivo* components of sini decoction and have cardiotonic activity by text mining.

^b^*In vivo* components of sini decoction and have cardiotonic activity through structural similarity comparison.

^c^*In vitro* components of sini decoction and have cardiotonic activity by text mining.
