# Supplementary material for: Drug target identification using network analysis: Taking active components in Sini decoction as an example
Source: Sci Rep. 2016 Apr 20;6:24245. doi: 10.1038/srep24245 (PMC4837341; doi:10.1038/srep24245)
Supplement: Supplementary Information [file srep24245-s1.pdf]

Supplementary Information

Drug target identification using network analysis: Taking active components in *Sini* decoction as an example

Si Chen[1], Hailong Jiang[1], Yan Cao[1], Yun Wang[1], Ziheng Hu[2], Zhenyu Zhu[1,*] & Yifeng Chai[1,*]

1 School of Pharmacy, Second Military Medical University, 325 Guohe Road, Shanghai, 200433, China

* Correspondence and requests for materials should be addressed to Z.Z. (email: zzyzyfzhu@163.com) or Y.C. (email: yfchai@smmu.edu.cn)

Supplementary Table S1. Metabolism of components in SND retrieving from admetSAR[3].

No.	Compounds	CYP450 1A2 inhibitor	CYP450 2C9 inhibitor	CYP450 2D6 inhibitor	CYP450 2C19 inhibitor	CYP450 3A4 inhibitor
**S3**	Mesaconine	No	No	No	No	No
**S4**	Senbusine A	No	No	No	No	No
**S5**	Senbusine B	No	No	No	No	No
**S6**	Talatizidine	No	No	No	No	No
**S7**	Aconine	No	No	No	No	No
**S8**	Hypaconine	No	No	No	No	No
**S9**	Fuziline	No	No	No	No	No
**S10**	Neoline	No	No	No	No	No
**S11**	Bikhaconine	No	No	No	No	No
**S12**	Talatisamine	No	No	No	No	No
**S13**	14-O-acetyl neoline	No	No	No	No	No
**S15**	Benzoylmes aconine	No	No	No	No	No
**S16**	Benzoylaco	No	No	No	No	No

	nine					
**S18**	Jesaconitine	No	No	No	No	No
**S19**	Beiwutine	No	No	No	No	No
**S21**	Mesaconitine	No	No	No	No	No
**S22**	Aconifine	No	No	No	No	No
**S30**	Crassicauline A	No	No	No	No	No
**S31**	Salsolinol	No	pAC50=4.8nM	No	No	No
**S32**	Coryneine	No	No	No	No	No
**S33**	Chuanfumine	No	No	No	No	No
**S34**	Karakanine	No	No	No	No	No
**Z14**	24-Hydroxy glycyrrhetic acid	No	No	No	No	pAC50=5nM
**Z25**	Methyl 3b, 24-dihygroxy olean-11,13(18)-diene 30-O ate	No	No	No	No	No
**Z27**	Betulic acid	No	No	No	No	No
**Z28**	Uralenolide	No	No	No	No	pAC50=5.4nM
**H27**	Licochalcone B	pAC50=7.2nM	No	No	pAC50=6.1nM	No
**H28**	Echinatin	pAC50=7.2nM	No	No	pAC50=6.1nM	No
**H29**	Isoliquiritigenin	pAC50=5.3nM	pAC50=4.7nM	pAC50=4.8nM	pAC50=4.9nM	pAC50=5.5nM
**H31**	Glepidotin	pAC50=5.45n	pAC50=5.6nM	pAC50=4.85nM	pAC50=5.55nM	pAC50=5.85nM

	C I	M

Reference

1 Cheng, F. *et al.* admetSAR: a comprehensive source and free tool for assessment of chemical ADMET properties. *J Chem Inf Model* **52**, 3099-3105, doi:10.1021/ci300367a (2012).

Supplementary Table S2. Results of the physiochemical characteristics similarity between 196 components in herbs in SND and 105 FDA-approved oral drugs from drugbank.

**Variables**	**Mean**		**Wilcoxon test**	**Kolmogorov-Smirnov test**
	drug	herb	Significance (2-tailed)[a]	Significance (2-tailed)[a]
**ALogP**	2.48556	3.03900	0.034	0.006
**Molecular Weight**	335.153013	425.179603	0.000	0.000
**Num_H_Acceptors**	4.12	6.60	0.000	0.000
**Num_H_Donors**	1.73	2.82	0.000	0.006
**Num-Rotatable Bonds**	5.97	7.32	0.003	0.020
**Num_AromaticRings**	1.54	1.08	0.005	0.025
**Molecular_Fractional/PolarSurfaceArea**	0.22856	0.23206	0.095	0.001

[a] The significance level is 0.05.

Supplementary Table S3. Targets of active components in SND validated by references.

**Gene symbol**	**Target name**	**Serial number**	**Name**	**Reference**	**Activity**
ESR2	Estrogen receptor beta	**H60**	Daidzein	[1]	Ki=300 nM
ESR1	Estrogen receptor alpha	**H60**	Daidzein	[1]	Ki=1800 nM
XDH	Xanthine dehydrogenase	**H60**	Daidzein	[2]	IC_{50}= 429800nM
PPARG	Peroxisome proliferator-activated receptor gamma	**H60**	Daidzein	[3]	Fold activation=1.2
ESR2	Estrogen receptor beta	**H56**	Davidigenin	[4]	IC_{50}= 1000000 nM
ESR1	Estrogen receptor alpha	**H56**	Davidigenin	[4]	IC_{50}= 1000000 nM
PPARG	Peroxisome proliferator-activated receptor gamma	**H28**	echinatin	[5]	Activity
PPARG	Peroxisome	**H34**	formononetin	[3]	Fold activation=

	proliferator-activated receptor gamma				4.6
XDH	Xanthine dehydrogenase	**H34**	formononetin	[6]	Inhibition= 17.6%
PPARG	Peroxisome proliferator-activated receptor gamma	**H41**	Gancaonin L	[5]	Activity
PPARG	Peroxisome proliferator-activated receptor gamma	**Z29**	Glycyrrhetic acid	[7]	Activity
PPARG	Peroxisome proliferator-activated receptor gamma	**H29**	Isoliquiritigenin	[3]	Fold activation= 1.3
XDH	Xanthine dehydrogenase	**H20**	Liquiritigenin	[6]	IC_{50}= 11300 nM
ESR1	Estrogen receptor alpha	**H10**	rutin	[8]	Activity=107.97 %
XDH	Xanthine dehydrogenase	**H10**	rutin	[9]	IC_{50}= 52200 nM
PPARG	Peroxisome proliferator-activated receptor gamma	**H10**	rutin	[10]	Activity
ADRA2A	Alpha-2A adrenergic receptor	**H10**	rutin	[11]	Ki=9340 nM
ESR1	Estrogen receptor alpha	**H11**	quercetin	[8]	Activity=76.81 %
HIF1A	Hypoxia-inducible factor 1-alpha	**H11**	quercetin	[12]	IC_{50}= 10200nM
PPARG	Peroxisome proliferator-activated receptor gamma	**H11**	quercetin	[3]	Fold activation= 1.3
CA2	Carbonic anhydrase II	**H11**	quercetin	[13]	Ki= 2540nM
XDH	Xanthine dehydrogenase	**H11**	quercetin	[14]	Fold activation=170
PON1	Serum paraoxonase/arylesterase 1	**H11**	quercetin	[15]	Activity
CDK2	Cyclin-dependent kinase 2	**H11**	quercetin	[16]	IC_{50}= 40000nM
ADRB2	Beta-2 adrenergic receptor	**S39**	higenamine	[17]	EC_{50}= 126.5 ng/mL
HMOX1	Heme oxygenase 1	**S39**	higenamine	[18]	Activity
NOS2	Nitric Oxide	**S39**	higenamine	[19]	IC_{50}=

	Synthase 2, Inducible				53μM
NOS2	Nitric Oxide Synthase 2, Inducible	**J13**	1-Dehydro-[6]-gingerdione	[20]	Activity
AGTR1	Angiotensin II Receptor, Type 1	**J54**	6-Gingerol	[21]	IC_{50}= 8.173 μM
ESR2	Estrogen receptor beta	**H20**	liquiritigenin	[22]	EC_{50}= 36.5 nM

Reference

1 Matsuda, H., Shimoda, H., Morikawa, T. & Yoshikawa, M. Phytoestrogens from the roots of Polygonum cuspidatum (Polygonaceae): structure-requirement of hydroxyanthraquinones for estrogenic activity. *Bioorg Med Chem Lett* **11**, 1839-1842 (2001).

2 Park, J. S., Park, H. Y., Kim, D. H., Kim, D. H. & Kim, H. K. ortho-dihydroxyisoflavone derivatives from aged Doenjang (Korean fermented soypaste) and its radical scavenging activity. *Bioorg Med Chem Lett* **18**, 5006-5009 (2008).

3 Matin, A. *et al.* 7-Hydroxy-benzopyran-4-one derivatives: a novel pharmacophore of peroxisome proliferator-activated receptor alpha and -gamma (PPARalpha and gamma) dual agonists. *J Med Chem* **52**, 6835-6850 (2009).

4 Zhao, L. & Brinton, R. D. Structure-based virtual screening for plant-based ERbeta-selective ligands as potential preventative therapy against age-related neurodegenerative diseases. *J Med Chem* **48**, 3463-3466 (2005).

5 Kuroda, M. *et al.* Phenolics from Glycyrrhiza glabra roots and their PPAR-gamma ligand-binding activity. *Bioorg Med Chem* **18**, 962-970 (2010).

6 Hayashi, T. *et al.* Inhibition of cow's milk xanthine oxidase by flavonoids. *J Nat Prod* **51**, 345-348 (1988).

7 Lallemand, B. *et al.* N-(2-{3-[3,5-bis(trifluoromethyl)phenyl]ureido}ethyl)-glycyrrhetinamide (6b): a novel anticancer glycyrrhetinic acid derivative that targets the proteasome and displays anti-kinase activity. *J Med Chem* **54**, 6501-6513 (2011).

8 Liu, L. *et al.* Discovery of estrogen receptor alpha modulators from natural compounds in Si-Wu-Tang series decoctions using estrogen-responsive MCF-7 breast cancer cells. *Bioorg Med Chem Lett* **22**, 154-163 (2012).

9 Cos, P. *et al.* Structure-activity relationship and classification of flavonoids as inhibitors of xanthine oxidase and superoxide scavengers. *J Nat Prod* **61**, 71-76 (1998).

10 Ha do, T. *et al.* The selected flavonol glycoside derived from Sophorae Flos improves glucose uptake and inhibits adipocyte differentiation via activation AMPK in 3T3-L1 cells. *Bioorg Med Chem Lett* **20**, 6076-6081 (2010).

11 Nahrstedt, A. & Butterweck, V. Lessons learned from herbal medicinal products: the example of St. John's Wort (perpendicular). *J Nat Prod* **73**, 1015-1021 (2010).

12 Ko, S., Lee, M. K., Shin, D. & Park, H. Structure-based virtual screening approach to the discovery of novel inhibitors of factor-inhibiting HIF-1: identification of new chelating groups for the active-site ferrous ion. *Bioorg Med Chem* **17**, 7769-7774 (2009).

13 Innocenti, A., Beyza Ozturk Sarikaya, S., Gulcin, I. & Supuran, C. T. Carbonic anhydrase inhibitors. Inhibition of mammalian isoforms I-XIV with a series of natural product

polyphenols and phenolic acids. *Bioorg Med Chem* **18**, 2159-2164 (2010).
14 Pauff, J. M. & Hille, R. Inhibition studies of bovine xanthine oxidase by luteolin, silibinin, quercetin, and curcumin. *J Nat Prod* **72**, 725-731 (2009).
15 Atrahimovich, D., Vaya, J. & Khatib, S. The effects and mechanism of flavonoid-rePON1 interactions. Structure-activity relationship study. *Bioorg Med Chem* **21**, 3348-3355 (2013).
16 Lee, J., Park, T., Jeong, S., Kim, K. H. & Hong, C. 3-Hydroxychromones as cyclin-dependent kinase inhibitors: synthesis and biological evaluation. *Bioorg Med Chem Lett* **17**, 1284-1287 (2007).
17 Bai, G. *et al.* Identification of higenamine in Radix Aconiti Lateralis Preparata as a beta2-adrenergic receptor agonist1. *Acta Pharmacol Sin* **29**, 1187-1194 (2008).
18 Ha, Y. M. *et al.* Higenamine reduces HMGB1 during hypoxia-induced brain injury by induction of heme oxygenase-1 through PI3K/Akt/Nrf-2 signal pathways. *Apoptosis* **17**, 463-474 (2012).
19 Kang, Y. J. *et al.* Inhibition of activation of nuclear factor kappaB is responsible for inhibition of inducible nitric oxide synthase expression by higenamine, an active component of aconite root. *J Pharmacol Exp Ther* **291**, 314-320 (1999).
20 Li, F. *et al.* In vitro antioxidant and anti-inflammatory activities of 1-dehydro-[6]-gingerdione, 6-shogaol, 6-dehydroshogaol and hexahydrocurcumin. *Food Chem* **135**, 332-337 (2012).
21 Liu, Q. *et al.* [6]-gingerol: a novel AT(1) antagonist for the treatment of cardiovascular disease. *Planta Med* **79**, 322-326 (2013).
22 Mersereau, J. E. *et al.* Liquiritigenin is a plant-derived highly selective estrogen receptor beta agonist. *Mol Cell Endocrinol* **283**, 49-57 (2008).

Supplementary Table S4. The significance of every relevant pathway of high degree (20-41), middle degree (10-19) and low degree (1-9) targets.

High Degree (20-41) pathway	p-value	Middle degree (10-19) pathway	p-value	Low degree (1-9) pathway	p-value
HIF-1 signaling pathway	1.95E-07	Dilated cardiomyopathy	6.46E-04	HIF-1 signaling pathway	5.48E-08
Calcium signaling pathway	9.49E-05	TNF signaling pathway	9.35E-04	VEGF signaling pathway	1.16E-07
Insulin secretion	8.32E-04	cGMP-PKG signaling pathway	2.03E-03	Oxytocin signaling pathway	5.53E-07
Oocyte meiosis	1.39E-03	Calcium signaling pathway	2.49E-03	cGMP-PKG signaling pathway	6.69E-07
Tuberculosis	3.59E-03	Neuroactive ligand-receptor interaction	5.84E-03	Calcium signaling pathway	1.22E-06
				Adrenergic signaling in cardiomyocytes	8.89E-06

Supplementary Table S5. In vivo components in SND retrieving from our previous research[1,2].

NO	In vivo components	Origins	Formula
**1**	Yunganoside K2	plasma and urine	C42H62O17
**2**	Uralsaponin B	urine	C42H62O16
**3**	Talatizamine	plasma and urine	C24H39NO5
**4**	Songorine	plasma and urine	C22H31NO3
**5**	Senbusine B	urine	C23H37NO6
**6**	Senbusine A	plasma and urine	C23H37NO6
**7**	Neoline	plasma and urine	C24H39NO6
**8**	Mesaconine	plasma and urine	C24H39NO9
**9**	Liquiritin apioside	plasma and urine	C26H30O13
**10**	Liquiritin	plasma and urine	C21H22O9
**11**	Liquiritigenin-O-sulfate	urine	C15H12O7S
**12**	Liquiritigenin glucuronide	plasma and urine	C21H21O10
**13**	Liquiritigenin	plasma and urine	C15H12O4
**14**	licoricesaponine H2/K2 or Uralsaponin B	plasma	C42H64O15
**15**	Licoricesaponin A3	plasma	C48H72O21
**16**	Licorice saponin J2	plasma	C42H64O16
**17**	Licorice saponin G2	plasma and urine	C42H62O17
**18**	Licorice saponin C2	plasma	C42H62O15
**19**	Licorice saponin B2	plasma	C42H64O15
**20**	Licorice saponin A3	urine	C48H72O21
**21**	licoisoflavone B-O-sulfate	urine	C20H16O9S
**22**	Licoisoflavone B	urine	C20H16O6
**23**	Licoisoflavone	urine	C20H18O6
**24**	Licochalcone D-O-glucuronide	urine	C27H30O11
**25**	Licochalcone D	urine	C21H22O5
**26**	Licobenzofuran-O-glucuronide	urine	C27H30O11
**27**	Licobenzofuran	urine	C21H22O5
**28**	Karakoline	urine	C22H35NO4
**29**	Karakolidine	urine	C22H35NO5
**30**	Isotalatizidine	plasma and	C23H37NO5

		urine	
**31**	Isomer of licorice saponin B2	plasma	C42H64O15
**32**	Isoliquiritin apioside	plasma and urine	C26H30O13
**33**	Isoliquiritin	plasma and urine	C21H22O9
**34**	Isoliquiritigenin-O-sulfate	urine	C15H12O7S
**35**	Isoliquiritigenin glucuronide	plasma and urine	C21H21O10
**36**	Isoliquiritigenin	plasma and urine	C15H12O4
**37**	Hypaconitine	urine	C33H45NO10
**38**	Hypaconine	urine	C24H39NO8
**39**	Hetisine	urine	C20H27NO3
**40**	Glyzaglabrin	urine	C16H10O6
**41**	Glycyrrhizin	plasma and urine	C42H62O16
**42**	Glycyrrhisoflvanone	urine	C21H20O6
**43**	Glycyrrhetic acid	plasma	C30H46O4
**44**	Glycyroside	plasma	C27H30O13
**45**	Glycycoumarin	urine	C21H20O6
**46**	Glucoliquiritin apioside	plasma	C32H40O18
**47**	Gancaonin N/Gancaonin B	urine	C21H20O6
**48**	Gancaonin L	urine	C20H18O6
**49**	Gancaonin B/Gancaonin N	urine	C21H20O6
**50**	Fuzitine	urine	C20H24NO4
**51**	Fuziline	plasma and urine	C24H39NO7
**52**	Foromonetin-O-sulfate	urine	C16H12O7S
**53**	Foromonetin-O-glucuronide	urine	C22H20O10
**54**	Foromonetin monohydroxylate	urine	C16H12O5
**55**	Foromonetin monohydroxylate	urine	C16H12O5
**56**	Foromonetin monohydroxylate	urine	C16H12O5
**57**	Foromonetin monohydroxylate	urine	C16H12O5
**58**	Foromonetin monohydroxylate	urine	C16H12O5
**59**	Formononetin glucuronide	plasma	C22H20O10
**60**	Formononetin	plasma and urine	C16H12O4
**61**	Dihydroformanonetin	urine	C16H14O4
**62**	Demethyl-8-methoxyl-14-benzoylaconine	urine	C32H45NO10
**63**	Demethyl-14-acetyltalatizamine	urine	C24H35NO7
**64**	Demethyl-14-acetylkarakoline	urine	C23H35NO5
**65**	Demethyl talatizamine	urine	C23H37NO5
**66**	Demethyl talatizamine	urine	C23H37NO5

**67**	Demethyl senbusine B	urine	C22H35NO6
**68**	Demethyl senbusine A	urine	C22H35NO6
**69**	Demethyl neoline	urine	C23H37NO6
**70**	Demethyl neoline	urine	C23H37NO6
**71**	Demethyl karakolidine	urine	C21H33NO5
**72**	Demethyl karakolidine	urine	C21H33NO5
**73**	Demethyl isotalatizidine	urine	C22H35NO5
**74**	Demethyl hypaconitine	urine	C32H43NO10
**75**	Demethyl dehydrogen isotalatizidine	urine	C22H33NO5
**76**	Demethyl benzoylhypaconine	urine	C30H41NO9
**77**	Demethyl benzoyldeoxyaconine	urine	C31H43NO9
**78**	Demethyl benzoylaconine	urine	C31H43NO10
**79**	Demethyl isotalatizidine	urine	C22H35NO5
**80**	Dehydrogen karakolidine	urine	C22H33NO5
**81**	Dehydrogen chuanfunine	urine	C22H33NO5
**82**	Dehydrated benzoylmesaconine	plasma and urine	C31H41NO9
**83**	Dehydrated benzoyhypaconine	urine	C31H41NO8
**84**	Dehydrated 6-gingerol	urine	C17H24O3
**85**	Davidigenin-O-sulfate	urine	C15H14O7S
**86**	Davidigenin-O-sulfate	urine	C15H14O7S
**87**	Davidigenin-O-sulfate	urine	C15H14O7S
**88**	Davidigenin-O-sulfate	urine	C15H14O7S
**89**	Davidigenin-O-glucuronide	urine	C21H22O10
**90**	Davidigenin	urine	C15H14O4
**91**	Daidzein-O-sulfate	urine	C15H10O7S
**92**	Daidzein	urine	C15H10O4
**93**	Chuanfunine	plasma and urine	C22H35NO5
**94**	Benzoylmesaconine	plasma and urine	C31H43NO10
**95**	Benzoylhypaconine	plasma and urine	C31H43NO9
**96**	Benzoyldeoxyaconine	plasma and urine	C32H45NO9
**97**	Benzoylaconine	plasma and urine	C32H45NO10
**98**	Beiwudine	urine	C31H41NO8
**99**	Aconine	plasma and urine	C25H41NO9
**100**	8-methoxyl-14-benzoylaconine	urine	C33H47NO10
**101**	6-Gingerol glucuronide	plasma	C23H34O10
**102**	6-Gingerol	urine	C17H26O4
**103**	4',7-dihydroxyflavone	urine	C15H10O4

**104**	16-β-Hydroxycardiopetaline	urine	C21H33NO4
**105**	14-benzoy-10-OH-mesaconine	urine	C31H43NO11
**106**	14-benzoy-10-OH-aconine	urine	C32H45NO11
**107**	14-acetyltalatizamine	plasma and urine	C26H41NO6
**108**	14-acetylneoline	urine	C26H41NO7
**109**	14-acetylkarakoline	urine	C24H37NO5
**110**	10-OH-mesaconine	urine	C24H39NO10
**111**	10-OH-aconine	urine	C25H41NO10
**112**	10-Hydroxytalatizamine	urine	C24H39NO6

Reference

1 Tan, G. *et al.* A strategy for rapid analysis of xenobiotic metabolome of Sini decoction in vivo using ultra-performance liquid chromatography-electrospray ionization quadrupole-time-of-flight mass spectrometry combined with pattern recognition approach. *J Pharm Biomed Anal* **96**, 187-196, doi:10.1016/j.jpba.2014.03.028 (2014).

2 Tan, G. *et al.* Analysis of phenolic and triterpenoid compounds in licorice and rat plasma by high-performance liquid chromatography diode-array detection, time-of-flight mass spectrometry and quadrupole ion trap mass spectrometry. *Rapid Commun Mass Spectrom* **24**, 209-218, doi:10.1002/rcm.4373 (2010).

Supplementary Table S6. Quality control statistics on the performance of text mining for the term "heart failure"

**Database Name**	**target proteins by searching**	**false positives**	**True target proteins**
**DrugBank database**	4	0	4
**OMIM**	2	1	1
**UniProtKB**	114	94	20
**TTD**	28	20	8
**GeneCards**	72	60	12

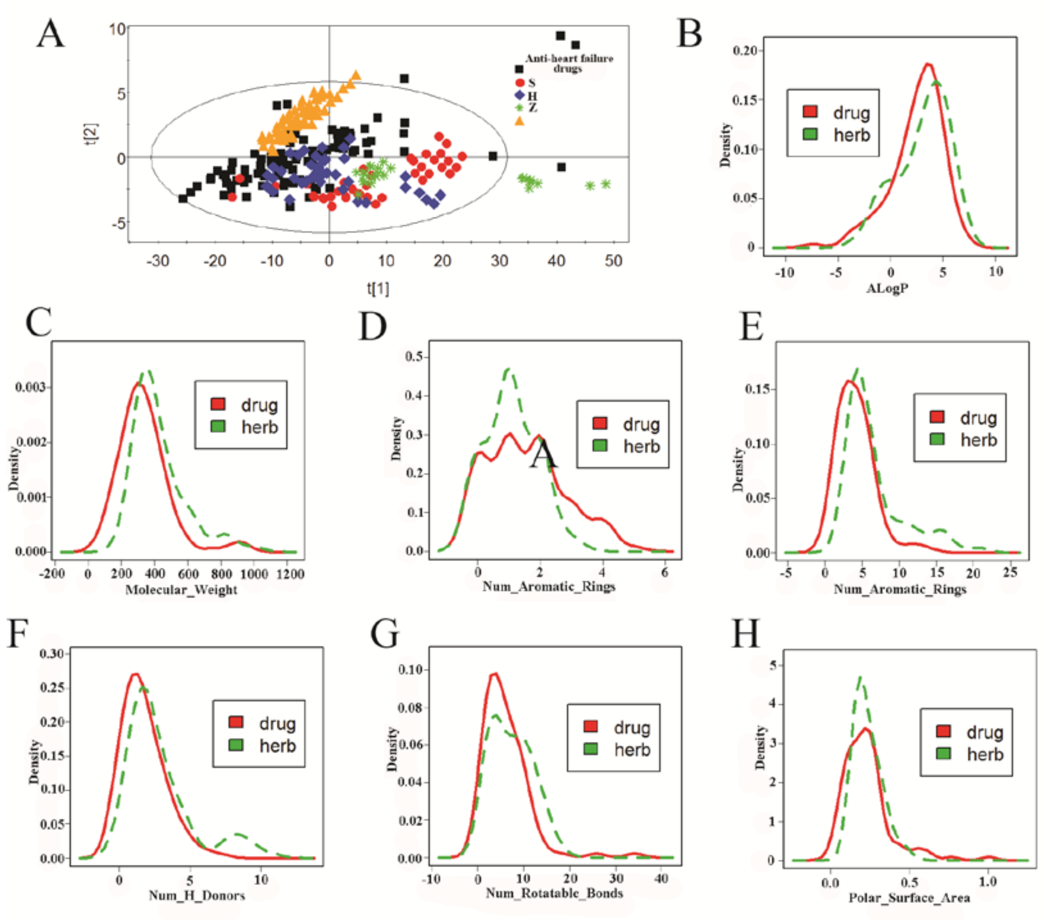

Supplementary Figure 1. Comparing chemical characteristics of active ingredients in SND. (A) PCA of active components in SND and approved anti-heart failure drugs calculated from seven chemical characteristics. Molecular weight; Num_AromaticRings: the number of aromatic rings; Num_H_Donors: the number of hydrogen bond donors; Molecular_FractionalPolarSurfaceArea: the molecular polar surface area; Num_RotatableBonds: the number of rotatable bonds; ALogP: the octanol–water partition coefficient; Num_H_Acceptors: the number of hydrogen bond acceptors. S: Aconitum carmichaelii, G: Glycyrrhiza uralensis, J: Zingiber officinale. (B)–(H) are distributions of these seven chemical characteristics of ingredients in SND and approved oral drugs.

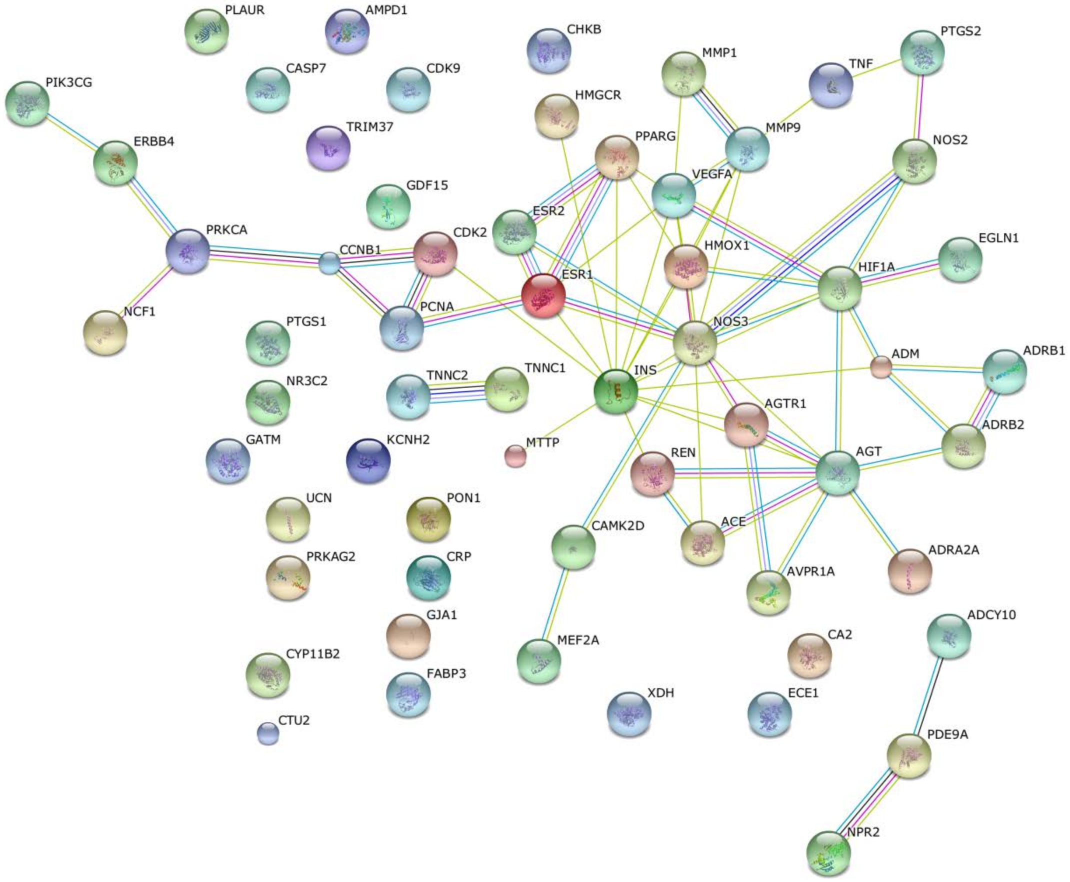

Supplementary Figure 2. Target protein-target protein interaction network
